# How the brain can be trained to achieve an intermittent control strategy for stabilizing quiet stance by means of reinforcement learning

**DOI:** 10.1007/s00422-024-00993-0

**Published:** 2024-07-12

**Authors:** Tomoki Takazawa, Yasuyuki Suzuki, Akihiro Nakamura, Risa Matsuo, Pietro Morasso, Taishin Nomura

**Affiliations:** 1 Graduate School of Engineering Science, Osaka University, 1-3 Machikaneyama, Toyonaka, Osaka 5608531 Japan; 2 Istituto Italiano di Tecnologia, Via Enrico Melen 83, Bldg B, 16152 Genoa, Italy; 3 Graduate School of Informatics, Kyoto University, Yoshida Honmachi, Sakyo-ku, Kyoto, 6068501 Japan

**Keywords:** Postural control, Postural sway, Intermittent control, Reinforcement learning

## Abstract

The stabilization of human quiet stance is achieved by a combination of the intrinsic elastic properties of ankle muscles and an active closed-loop activation of the ankle muscles, driven by the delayed feedback of the ongoing sway angle and the corresponding angular velocity in a way of a delayed proportional (P) and derivative (D) feedback controller. It has been shown that the active component of the stabilization process is likely to operate in an intermittent manner rather than as a continuous controller: the switching policy is defined in the phase-plane, which is divided in dangerous and safe regions, separated by appropriate switching boundaries. When the state enters a dangerous region, the delayed PD control is activated, and it is switched off when it enters a safe region, leaving the system to evolve freely. In comparison with continuous feedback control, the intermittent mechanism is more robust and capable to better reproduce postural sway patterns in healthy people. However, the superior performance of the intermittent control paradigm as well as its biological plausibility, suggested by experimental evidence of the intermittent activation of the ankle muscles, leaves open the quest of a feasible learning process, by which the brain can identify the appropriate state-dependent switching policy and tune accordingly the P and D parameters. In this work, it is shown how such a goal can be achieved with a reinforcement motor learning paradigm, building upon the evidence that, in general, the basal ganglia are known to play a central role in reinforcement learning for action selection and, in particular, were found to be specifically involved in postural stabilization.

## Introduction

**Fig. 1:**
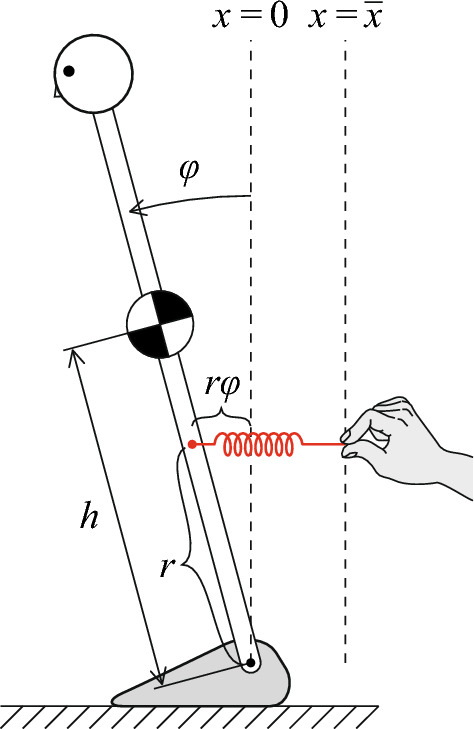
An inverted pendulum as a model of the upright stance. In Eq. (1), *I* is the inertia moment of the pendulum around its pin-jointed distal end that corresponds to the ankle joint for a standing body, *g* the gravity acceleration, *m* the mass of the pendulum, and *h* the distance from the joint to the center of mass (CoM) of the pendulum. $\varphi$ is the tilt angle of the pendulum from the upright position, where $\varphi >0$ represents the forward tilt. In the right-hand-side of Eq. (1), the first term $mgh\varphi$ represents the linearized gravitational toppling torque of $mgh\sin \varphi$ for a small range of motion $\varphi$ during quiet stance, followed by the toppling-force-resistant force, which is generated by a simple linear spring that is placed horizontally. One end of the spring is attached to the pendulum near the joint. The other end is located at $x=\bar{x}$, which is held by fingers of a man who manually keeps balance of the pendulum. $k_p$ and *b* represents the spring constant of the spring and *b* the viscous coefficient of the torsional viscosity of the ankle joint, respectively. $\ell _0$ and *r* are the natural length of the spring and the moment arm for the spring-based actuation for the pendulum, respectively

Understanding mechanisms of how we maintain upright stance offers profound insights into the information processing in the brain for handling precarious nature of body movements under the influence of destabilizing factors, including gravity, sensory feedback delay and motor noise (Rasman et al. 2024). Simple inverted pendulum models, moving in the sagittal plane for describing postural sway in the anterior-posterior direction, have been playing a pivotal role in the study of neural control during quiet stance (Morasso et al. 2019). Such a model can be formulated as

1 $$\begin{aligned} I\ddot{\varphi }=mgh\varphi -rk_p\left( \bar{x}+r\varphi -\ell _0 \right) -b\dot{\varphi }\end{aligned}$$

where $\varphi$ is the small tilt angle of the pendulum from the upright position (Loram et al. 2005) (see Fig. 1 for details). Despite the fanciful setting of Fig. 1 with a fingertip-based manual control of the pendulum, there are clear correspondences between this caricature and the human postural control system: the spring and the horizontal position of the fingertips represent, respectively, the Achilles tendon and the degree of activation of active contractile element of the calf muscles that are connected in series with each other. Using this setting, we begin this article by highlighting a core issue to be addressed in the study of postural control.

It has been long assumed that a hard-spring-like property of the Achilles tendon is a key mechanism of how the quiet stance at the upright position is stabilized (Fitzpatrick et al. 1992; Winter et al. 1998). Namely, it has been believed that the spring of the Achilles tendon is so hard (the stiffness $k_p$ is so large) that the upright posture of the pendulum can be maintained simply by placing the fingertips at an appropriate fixed horizontal position $\bar{x}$, where $\bar{x}$ corresponds to the tonus of the calf muscles as the anti-gravity plantar flexor muscles: placing the fingertips more right and left, respectively, correspond to the larger and smaller degrees of the muscle tonus determined by the brain in a feedforward manner (Gatev et al. 1999). Since this model has only one muscle-tendon actuator for plantar flexion, which is based on the fact that the tibialis anterior muscle as a dorsiflexor is quite less frequently activated during quiet stance (Masani et al. 2003), a stabilization of the pendulum would be achieved only at a slightly forward-tilted equilibrium posture, where the gravitational toppling torque and the plantar-flexion torque generated by the restoring force of the spring are balanced. Note that the plantar-flexion torque generated by the spring should be equal to the active force generated by the calf muscles, because they are connected with each other in series. The major issue of the postural control research is stability of the equilibrium and mechanisms of how it is achieved.

The forward-tilted equilibrium causes a tendency of the body to forward fall, due to gravitational toppling torque (Sakanaka et al. 2021). For this reason, stochastic postural sway in anterior–posterior direction during quiet stance tends to exhibit a repetition of forward *micro-fall* and the subsequent backward *micro-recovery* (Loram et al. 2005; Nakamura et al. 2023). The traditional theory states that the muscle tonus is kept unchanged basically in response to each micro-fall (Horak and Macpherson 2011), i.e., the position of the fingertips $\bar{x}$ in Eq. (1) is fixed. For the fixed $\bar{x}$, it is apparent by the geometrical consideration that the total length of the tendon-muscle system (a distance between the proximal end of the muscle and the fingertips in Fig. 1) is necessarily elongated by a forward tilt of the pendulum during each micro-fall. Because the tendon with the large stiffness is hardly elongated, the amount of elongation of the total length of the tendon-muscle system is mostly attributed to the stretch of the calf muscles (Horak and Macpherson 2011). The traditional theory claims that a restoring force generated by the stretch of the calf muscles (basically with its tonus unchanged) pulls the pendulum backward to achieve the micro-recovery. If the stiffness of the calf muscles is inadequate for the micro-recovery, the resistance to stretch of the calf muscles may be augmented by stretch reflexes or by central control (Loram et al. 2005), i.e., by shifting $\bar{x}$ rightward.

Recent studies by Loram et al. (2005), however, revealed a paradoxical phenomenon contradicting the traditional theory. That is, the calf muscles are shortened and the Achilles tendon is elongated during each micro-fall, which means that the spring constant $k_p$ of Achilles tendon is much smaller than that has long been believed. In other words, the restoring force necessary for the micro-recovery cannot be generated by the stretch of the calf muscles, neither by the stretch-reflex-based augmentation of the resistance to stretch of the calf muscles, because of the absence of the stretch of the calf muscles, which has been considered as the primary cause of the restoring force generation. After the discovery that challenges the traditional theory, the community of postural control research is awaiting a new theory of postural control during quiet stance (Morasso and Schieppati 1999; Morasso and Sanguineti 2002; Loram et al. 2011). One certain thing is that a new theory requires an appropriate mechanism for active modulation of $\bar{x}$ without the help of the stretch reflex of calf muscles. If not, the inverted position of the pendulum cannot be stabilized by any fixed $\bar{x}$ (Morasso and Sanguineti 2002).

We make the situation more specific using the model of Eq. (1) and Fig. 1. Note that, in Eq. (1), $\ell _0$ and $\bar{x}+r\varphi$ represent the natural and the actual lengths of the spring, respectively, where $\bar{x}$ is the horizontal position of the fingertips from the origin located at the joint, and *r* is the moment arm for the spring-based actuation for the pendulum (Fig. 1). As in the traditional theory, we consider a simpler case with a fixed $\bar{x}$, i.e., a case with no stretch reflex nor any central modulation of $\bar{x}$. Defining $k\triangleq r^2k_p$, $\bar{\alpha }\triangleq \bar{x}/r$, $\varphi _0\triangleq \ell _0/r$, and $\varphi _{\text {EP}}\triangleq \varphi _0-\bar{\alpha }$, we rewrite Eq. (1) as

2 $$\begin{aligned} I\ddot{\varphi }=mgh\varphi -k\left( \varphi -\varphi _{\text {EP}} \right) -b\dot{\varphi }, \end{aligned}$$

where *k* is the passive torsional spring constant of the joint (passive joint stiffness). $\varphi _{\text {EP}}$ can be regarded as the *virtual equilibrium point* (VEP) that would be followed by an actual trajectory of $\varphi$, as often discussed in literatures of voluntary arm reaching movements (Hogan 1985; Gomi and Kawato 1997; Loram et al. 2005). $\varphi _{\text {EP}}=0$ when $\bar{x}=\ell _0$. $\varphi _{\text {EP}}<0$ when the fingertips are located at the right side of $x=\ell _0$, i.e., $\bar{x}>\ell _0$, which makes $\varphi -\varphi _{\text {EP}}$, the deviation of $\varphi$ from the VEP, and the resulting restoring force large. On the other hand, $\varphi _{\text {EP}}>0$ when the fingertips are located at the left side of $x=\ell _0$, i.e., $\bar{x}<\ell _0$, which makes $\varphi -\varphi _{\text {EP}}$ and the resulting restoring force small. Note that $\bar{x}+r\varphi >\ell _0$ should always be satisfied by the ankle muscles, as the tendon is always stretched compared to the natural length (Horak and Macpherson 2011), and thus $\varphi >\varphi _{\text {EP}}$, which means that the spring of the tendon in Fig. 1, and also in the actual Achilles tendon in human, can work only as a tension spring, not as a compression spring. In contrast to the restoring torque $-k(\varphi -\varphi _{\text {EP}})$ that pulls the pendulum backward in proportion to $\varphi >0$, the term $mgh\varphi$, as a linearized version of the gravitational toppling torque $mgh\sin \varphi$ for small $\varphi$, is an anti-restoring torque, i.e., it pulls the pendulum forward in proportion to $\varphi$. The critical constant *mgh* is often referred to as the *load stiffness* (Chew et al. 2008).

Identification of the value of *k* in comparison with the value of *mgh* is critical for the study of postural control (Loram and Lakie 2002a; Casadio et al. 2005). Defining $k\triangleq c\cdot mgh$ with *c* representing the ratio between the passive joint stiffness and the load stiffness as in Loram et al. (2005), we have a difference between two competing torques denoted by

3 $$\begin{aligned} \tau \triangleq mgh\varphi -c\cdot mgh(\varphi -\varphi _{\text {EP}}) =mgh \left\{ (1-c)\varphi +c\varphi _{\text {EP}} \right\} .\nonumber \\ \end{aligned}$$

$\tau =0$ when two forces are balanced, yielding the equilibrium posture at

4 $$\begin{aligned} \bar{\varphi }=\frac{c}{c-1}\varphi _{\text {EP}}. \end{aligned}$$

Note that $\bar{\varphi }$ becomes closer to $\varphi _{\text {EP}}$ of the VEP as *c* increases over unity for large values of *k*. With $\varphi =\bar{\varphi }+\theta$ for a small deviation $\theta$ from the equilibrium $\bar{\varphi }$, Eqs. (2) and (3) are rewritten as

5 $$\begin{aligned} \tau =mgh(1-c)\theta \end{aligned}$$

and

6 $$\begin{aligned} I\ddot{\theta }=mgh(1-c)\theta -b\dot{\theta }, \end{aligned}$$

respectively. If $c>1$ as assumed in the traditional theory with a hard spring of the Achilles tendon, a forward-tilted equilibrium is achieved only with $\varphi _{\text {EP}}>0$, by locating the fingertips at the left side of $x=\ell _0$. Apparently, the equilibrium posture $\bar{\varphi }$ or $\theta =0$ is stable for $c>1$ (Winter et al. 1998). On the other hand, if $c<1$ with a compliant spring, a forward-tilted equilibrium is achieved only with $\varphi _{\text {EP}}<0$, by locating the fingertips at the right side of $x=\ell _0$, corresponding to the large muscle tonus $x=\bar{x}$, perhaps with a large elongation of the compliant spring. The equilibrium posture $\bar{\varphi }$ or $\theta =0$ for $c<1$ is unstable, no matter how much the muscle tonus $\bar{x}$ is set to a large value. That is, contrary to intuitive expectations at first glance, the upright posture with the compliant Achilles tendon can never be stabilized by elevating the tonus of the calf muscles. Note that the unstable equilibrium point in the state space of the system is topologically saddle point with a stable eigenmode along a stable manifold of the saddle and an unstable eigenmode along an unstable manifold of the saddle. It might also be counter-intuitive that the restoring torque $-k(\varphi -\varphi _{\text {EP}})$ can be balanced with the gravitational toppling torque even with a small value of *k*, which can be achieved by making a deviation of $\varphi$ from the VEP large. That is, there exists an equilibrium posture $\bar{\varphi }$ in the system even with a small value of *k* with $c<1$, although the equilibrium posture cannot be stable with a fixed value of the tonus $\bar{x}$. That is, static equilibration and dynamic stabilization of the equilibrium are not the same thing.

Reliable quantifications of the *k* value during quiet stance were performed relatively recently (Loram and Lakie 2002a; Casadio et al. 2005), showing that $k<mgh$, i.e., $c<1$. That is, the passive joint stiffness is smaller than the load stiffness ($c\sim 0.8$), and thus insufficient for stabilizing the upright posture by elevating the tonus of the calf muscles. It is also noteworthy to mention that the passive joint viscosity *b* is also small, about a few Nms/rad (Loram and Lakie 2002a; Casadio et al. 2005), corresponding to the underdamped situation. Based on the above discussion, only one remaining option to avoid a fall of the pendulum is to alter the value of $\varphi _{\text {EP}}$ actively, i.e., to move the position of the fingertips $\bar{x}$ actively, perhaps by using the sensory feedback information (Masani et al. 2003) and/or a predicted state of the pendulum (Gawthrop et al. 2011), or by using an appropriate periodic control force (Insperger 2006; Insperger and Milton 2014). With a dynamic alteration of $\bar{x}$ by an additional amount of $\tilde{x}(t)$, the length of the spring becomes $\bar{x}+\tilde{x}(t)+r\varphi$, and Eq. (2) for $c<1$ is rewritten as

7 $$\begin{aligned} I\ddot{\theta }=mgh(1-c)\theta -b\dot{\theta }+k\tilde{\varphi }_{\text {EP}}(t), \end{aligned}$$

where $\tilde{\varphi }_{\text {EP}}(t)\triangleq -\tilde{x}(t)/r$, for which we use the fact that the motion of the fingertips does not affects the viscous force around the joint. The last term $k\tilde{\varphi }_{\text {EP}}(t)$ represents the active modulation of the VEP, corresponding to the active control torque generated by a sequence of phasic contractions of the calf muscles, which is superposed on the tonic contraction of the calf muscles $\bar{\alpha }$.

Defining $\tau _{\text {act}}(t)\triangleq k\tilde{\varphi }_{\text {EP}}(t)$, we aim to reveal a neural controller for $\tau _{\text {act}}$ that is consistent with experimental characterizations of postural sway, including the non-spring-like paradoxical behavior of the calf muscles during micro-fall and the subsequent micro-recovery. That is, the controller would generate active torque to brake each micro-fall, corresponding to the contraction of the calf muscles during the micro-fall, and make the calf muscles relaxed, i.e., switching the active force generation off during the micro-recovery. Note that, in this case, the micro-recovery in the absence of the active torque $\tau _{\text {act}}$ might be achieved passively by the inertia force of the pendulum that is thrown backward with a negative velocity at the timing of when $\tau _{\text {act}}$ is switched off (Loram and Lakie 2002b). The promising candidate for such a controller, referred to as the intermittent control model (Bottaro et al. 2008; Asai et al. 2009), may be formulated by

8 $$\begin{aligned} \tau _{\text {act}}(t)=\left\{ \begin{array}{ll} -P\theta _\Delta -D\omega _\Delta , &{}\quad \text{ if } (\theta _\Delta ,\omega _\Delta )^T\in \text {S}_{\text {on}}, \\ 0, &{}\quad \text{ otherwise } \text{ if } (\theta _\Delta ,\omega _\Delta )^T\in \text {S}_{\text {off}}, \end{array} \right. \end{aligned}$$

where $\theta _{\Delta }\triangleq \theta (t-\Delta )$ and $\omega _{\Delta }\triangleq \omega (t-\Delta )\triangleq \dot{\theta }(t-\Delta )$, representing the delay-affected tilt angle and the angular velocity with $\Delta$ being the feedback delay time due to neural signal transmissions.

**Fig. 2:**
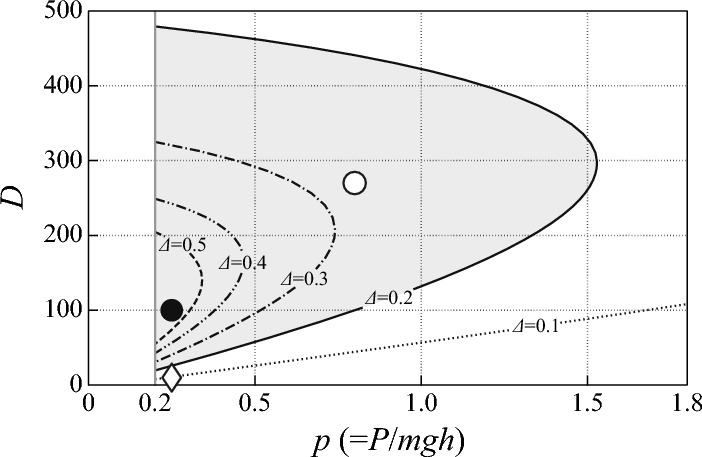
Stability regions of the on-subsystem for the intermittent control model on *P*–*D* parameter plane for several values of delay $\Delta = 0.1$, 0.2, 0.3, 0.4 and 0.5 s. Stability region for $\Delta =0.2$ s is indicated by the gray area. The on-subsystem is stable for the parameter points at $(P,D)=(0.25mgh,100)$ with the closed circle and at $(P,D)=(0.8mgh,270)$ with the open circle, while it is unstable for the parameter points at $(P,D)=(0.25mgh,10)$ with the diamond. The parameter point $(P,D)=(0.8mgh,270)$ is shown here as a reference that is close to the one used for the traditional continuous control model

The parameters *P* and *D* are gains of the conventional proportional and derivative controller, respectively, when $\tau _{\text {act}}(t)$ is activated. Note that, for small values of $\theta$ and $\omega$, it is quite natural to consider the PD controller for the generation of the active joint torque, by taking into account a Taylor expansion of a general feedback torque with any form of smooth nonlinear function of $\theta _\Delta$ and $\omega _\Delta$. For the intermittent control model with $\tau _{\text {act}}$ in Eq. (8), the $\theta$–$\omega$ plane is divided into two regions, $\text {S}_{\text {on}}$ and $\text {S}_{\text {off}}$, in which the active torque is switched on and off, respectively, in a delayed-state-dependent manner. Namely, the PD controller is activated during the period of time when the delayed state $(\theta _\Delta ,\omega _\Delta )$ is located in $\text {S}_{\text {on}}$, while it is inactivated during the period of time when $(\theta _\Delta ,\omega _\Delta )$ is in $\text {S}_{\text {off}}$. The intermittent control model can be viewed as a stochastic *switching hybrid dynamical system*, alternating between the on-subsystem (the system with the PD controller switched on persistently) described by

9 $$\begin{aligned} I\ddot{\theta }=mgh\theta -k\theta -b\dot{\theta }-P\theta _\Delta -D\omega _\Delta +\sigma \xi (t) \end{aligned}$$

for $(\theta _\Delta ,\omega _\Delta )\in \text {S}_{\text {on}}$, referred to as the on-region, and the off-subsystem described by

10 $$\begin{aligned} I\ddot{\theta }=mgh\theta -k\theta -b\dot{\theta }+\sigma \xi (t) \end{aligned}$$

for $(\theta _\Delta ,\omega _\Delta )\in \text {S}_{\text {off}}$, referred to as the off-region. The last terms introduced here in the right-hand-sides of Eqs. (9) and (10) are the process noise (the endogenous torque noise) with $\xi (t)$ and $\sigma$ being the standard Gaussian white noise and its standard deviation, respectively. Traditionally, this sort of noise was the only source of postural sway, where relatively large noise intensities, tens of Newton meter per radian, have been assumed for modeling postural sway (Peterka 2002; Maurer and Peterka 2005). On the other hand, the intermittent control model can exhibit realistic postural sway with very small noise, or even with no noise, because of the existence of off-subsystem (Bottaro et al. 2008; Asai et al. 2009).

Note that the on-subsystem of Eq. (9) is a described by a delay differential equation (DDE). One of the critical factors that determines dynamics of the intermittent control model with a given time-delay $\Delta$ is stability of the DDE on-subsystem, which can be characterized by the stability region in the *P*–*D* parameter plane (Fig. 2). The stability region of the deterministic on-subsystem with $\sigma =0$ in the *P*–*D* parameter space is D-shaped (Insperger et al. 2015; Suzuki et al. 2020) with the vertical boundary of “D” located at $P=(1-c)mgh$ for $c<1$ (Fig. 2), which is equal to 0.2*mgh* for $c=0.8$. In this case, the on-subsystem is unstable for the *P*–*D* values located in the outer region of the D-shpaed stability region. Particularly, instability at the curved line boundary of D-shaped stability region is due to the delay-induced instability in the delay feedback control system of Eq. (9). The larger the delay $\Delta$, the smaller is the D-shaped stability region (Fig. 2).

**Fig. 3:**
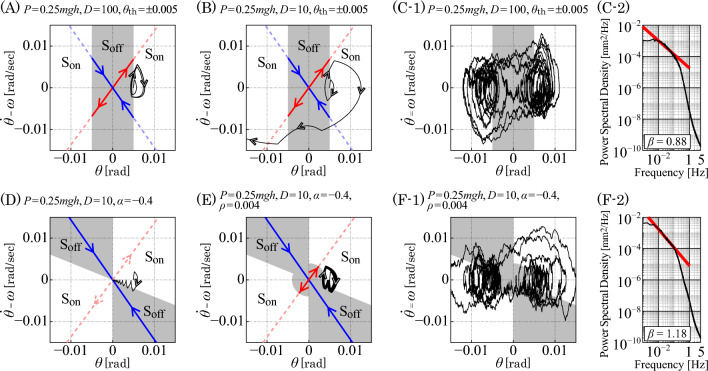
Comparisons between delay threshold control model and the intermittent control model. **A**–**C** A delay threshold control model with $(P,D)=(0.25mgh,100)$ for a stable on-subsystem, as indicated by the closed circle in Fig. 2. **D**–**F** The intermittent control model with $(P,D)=(0.25mgh,10)$ for an unstable on-subsystem, as indicated by the diamond in Fig. 2. $S_{\text {off}}$. $\text {S}_{\text {on}}$ and $\text {S}_{\text {off}}$ are separated by the boundaries defined by $\theta _\Delta =0$, $\omega _\Delta =\alpha \theta _\Delta$ and $\theta _\Delta ^2+\omega _\Delta ^2=\rho ^2$. The parameter $\alpha$ determines the slope of the on–off boundary line $\omega _\Delta =\alpha \theta _\Delta$. The parameter $\rho$ determines the small circular sensory dead zone around the upright position. Panels **C-1**, **C-2** and **F-1** and **F-2** are for the stochastic dynamics with noise ($\sigma =0.2$ Nm/rad)

Different spatial configurations of $\text {S}_{\text {on}}$ and $\text {S}_{\text {off}}$ on the $\theta$–$\omega$ plane lead to different sway dynamics and stability of the intermittent control model. Representatives of such models are the one with a threshold strategy (Collins and De Luca 1994; Eurich and Milton 1996; Nema et al. 2015) and the one that exploits the stable manifold (the stable eigenmode) of the unstable saddle point of Eq. (10) for the off-subsystem (Bottaro et al. 2008; Asai et al. 2009). The threshold strategy simply takes into account the sensory dead zone, i.e., the existence of a range of sensory input in which no corrective actions are taken (Eurich and Milton 1996; Insperger and Milton 2017). On the other hand, the intermittent control that exploits the stable eigenmode of the off-subsystem might be predicated on a strategy for action selections in the brain (Nakamura et al. 2021, 2023; Michimoto et al. 2016). In this sequel, the name of the intermittent control model is reserved for the model with the controller of Eq. (8) that exploits the stable manifold of the off-subsystem, whereas the model with the simple threshold is simply referred to as the (delay) threshold control model. Figure 3 compares deterministic and stochastic dynamics of the delay threshold control model and the intermittent control model. The delay threshold control model, for which $\text {S}_{\text {off}}$ is defined by upper and lower thresholds of the tilt angle $\theta$ (two vertical threshold lines at $\pm \, \theta _{\text {th}}$ in the $\theta$–$\omega$ plane) sandwiched by two $\text {S}_{\text {on}}$ regions, can stabilize the inverted pendulum, when (and probably only when) the on-subsystem is stable, as shown in Fig. 3A. A stochastic version of the threshold control model exhibits a switching between oscillatory dynamics near the right and the left boundaries of $\text {S}_{\text {off}}$ as in Fig. 3C-1, with its power spectral density (PSD) function for the time series of $\theta (t)$ as shown in Fig. 3C-2. Since the stabilizer of the system in this case is the stable on-subsystem, the upright posture of the inverted pendulum with the delay threshold controller cannot be stable, when the on-subsystem is unstable with the *P*–*D* parameters located outside of the D-shaped stability region, particularly for small values of *P* and *D* of Eq. (9), leading to a fall as shown in Fig. 3B. In contrast to the threshold control model, the intermittent control model, for which $\text {S}_{\text {off}}$ is arranged to cover the stable manifold of the off-subsystem in the second and fourth quadrant of the $\theta$–$\omega$ plane, can stabilize the inverted pendulum even with small values of *P* and *D* for the unstable off-subsystem. This is made possible by repeated use of the contractive dynamics associated with the stable eigenmode of the off-subsystem as shown in Fig. 3D and E for the deterministic dynamics. Figure 3F-1 shows stochastic dynamics corresponding to Fig. 3E, for which the PSD exhibits a power-law-like property in the low-frequency band between $10^{-3}$ and $10^{-1}$ Hz with the scaling exponent $\beta \sim 1$, as shown in Fig. 3F-2. Note that the power-law scaling exponent $\beta$ in the low-frequency band was evaluated using a least square linear fit for the frequency range of [0.004–0.1] Hz throughout the paper in this sequel. The comparisons illustrated in Fig. 3 demonstrate that the intermittent control model is more robust against changes in the *P*–*D* parameter values, compared to the threshold control model. Moreover, because the on-subsystem for the intermittent control model can bring the state point in $\text {S}_{\text {on}}$ to $\text {S}_{\text {off}}$ regardless of whether it is unstable or stable on-subsystem, the intermittent control model is more robust also against the feedback delay-time $\Delta$, compared to the threshold control model.

Evidence is accumulating to support the intermittent control model as the mechanism of human postural control for stabilizing quiet stance (e.g., Perera et al. 2018; Xiang et al. 2018; Tanabe et al. 2017; Tietavainen et al. 2017; McKee and Neale 2019; Suzuki et al. 2020; Nakamura et al. 2023; Tigrini et al. 2000). Particularly, we showed recently that the intermittent control model can better fit postural sway data from healthy young adults, compared to the stiffness control model (i.e., the model without $\text {S}_{\text {off}}$), using a technique of Bayesian parameter inference for the model (Suzuki et al. 2020). Interestingly, postural sway data from patients with Parkinson’s disease exhibiting severe postural symptoms can be better fitted by the model with less intermittency, i.e., by the model with no or a very small off-region $\text {S}_{\text {off}}$, suggesting that the appropriate placement of the off-region $\text {S}_{\text {off}}$ is critical for the postural stabilization. Moreover, based on the fact that the basal ganglia is responsible for the cause of postural instability in patients with Parkinson’s disease, we can speculate that information processing performed by the basal ganglia might be associated with the determination of on- and off-regions for the intermittent controller.

The purpose of this study is to provide insights into how the on–off switching-type state-dependent feedback controller of the intermittent control model can be established in the brain for stabilizing quiet stance. Multiple lines of evidence suggest that the basal ganglia are involved in the postural stabilization as well as in the postural instability in patients with Parkinson’s disease (Takakusaki et al. 2003; Perera et al. 2018; Yamamoto et al. 2011). Moreover, it has been considered that the basal ganglia play a central role in reinforcement learning for action selection (Doya 2000; Bostan and Strick 2018). Taken together, we hypothesize that an appropriate state-dependent selections of “on” and “off” of the active feedback control during quiet stance is acquired through reinforcement learning. In this study, we explore an instantaneous reward/cost function, whose cumulative values lead to the intermittent control strategy that exploits the stable eigenmode of the saddle of the off-subsystem using the unstable oscillation of the on-subsystem with small *P*–*D* feedback gains.

## Materials and methods

In this study, the on-subsystem as a DDE with Eq. (9) is approximated by the following ordinary differential equation (ODE):

11 $$\begin{aligned} (I-D\Delta )\ddot{\theta }+(b+D-P\Delta )\dot{\theta }+(k+P-mgh)\theta =\sigma \xi , \end{aligned}$$

which is obtained using the Taylor expansions of $\theta (t-\Delta )\approx \theta (t)-\dot{\theta }(t)\Delta$ and $\dot{\theta }(t-\Delta )\approx \dot{\theta }(t)-\ddot{\theta }(t)\Delta$ for a small $\Delta$. It has been confirmed that this ODE approximation of the original DDE works satisfactory for the current control system (Stepan and Kollar 2000), even for stochastic dynamics in the presence of process noise (Suzuki et al. 2023). However, note that stability regions of the delayed system in Fig. 2 and the stability region of the non-delayed ODE system of Eq. (11) are different to a certain extent (Insperger 2015).

The use of this ODE approximation is to avoid issues arising from the feedback delay in reinforcement learning (Nath et al. 2021). Related studies that deal with reinforcement learning for the model with the DDE on-system will be presented elsewhere. Values of the parameters in the model were set as summarized in Table 1 in Appendix, according to the previous studies (Winter et al. 1998; Peterka 2002). The state space representation of Eq. (11) is written as

12 $$\begin{aligned} \frac{d}{dt}\begin{pmatrix} \theta \\ \omega \end{pmatrix}=\begin{pmatrix} 0 &{}\quad 1 \\ -\frac{k+P-mgh}{I-D\Delta } &{}\quad -\frac{b+D-P\Delta }{I-D\Delta } \end{pmatrix}\begin{pmatrix} \theta \\ \omega \end{pmatrix}+\begin{pmatrix} 0 \\ \tilde{\sigma }\xi \end{pmatrix}, \end{aligned}$$

where $\tilde{\sigma }=\sigma /(I-D\Delta )$. Defining the state vector as $x\triangleq (\theta \; \, \omega )^T$, the linear stochastic differential equation of Eq. (12) is denoted formally by

13 $$\begin{aligned} \frac{dx}{dt}=A_\Delta (P,D)x+\tilde{\Sigma }(D)\xi , \end{aligned}$$

with $\tilde{\Sigma }\triangleq (0\; \; \tilde{\sigma })^T$. For a given set of gain parameters *P* and *D*, including the case with $P=D=0$ for the off-subsystem, with an initial state $x_0$, the deterministic flow $\phi (x_0,t)$ of Eq. (13) with $\sigma =\tilde{\sigma }=0$ is expressed analytically as

14 $$\begin{aligned} \phi (x_0,t;P,D)=\exp \left( A_\Delta (P,D)t \right) x_0. \end{aligned}$$

We consider dynamics of a time-discretized version of Eq. (13), in which dynamics of the model between two consecutive time instants are represented by the analytical solution of Eq. (14). That is, a state transition governed by Eq. (14) from $x_n$ at the discrete time *n* to $x_{n+1}$ at the next time $(n+1)$ for a small time step $\delta t$ is given by

15 $$\begin{aligned} x_{n+1}=\phi (x_n,\delta t;P,D), \end{aligned}$$

The effective intensity of noise to determine a stochastic state transition for Eq. (14) is obtained based on the following Euler–Maruyama discretization of Eq. (13):

16 $$\begin{aligned} x_{n+1}=\left[ I+\delta t A_\Delta (P,D)\right] x_{n}+\hat{\Sigma }\xi _{n}, \end{aligned}$$

where $\hat{\Sigma }\triangleq (0\; \; \hat{\sigma })^T\triangleq \sqrt{\delta t}\tilde{\Sigma }$. Using the fact that the Taylor expansion of Eq. (15) is

17 $$\begin{aligned} \phi (x_n,\delta t; P,D) \simeq \left[ I+\delta t A_\Delta (P,D)\right] x_n, \end{aligned}$$

we approximate the stochastic state transition of the system by
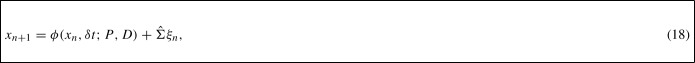


which is used as a basis for computing the state transition probability matrix as described later, instead of using Eq. (16). The use of Eq. (18) provides a better approximation of Eq. (13) with a relatively large size of the time step $\delta t$. In this study, the time step $\delta t=0.01$ s was used as the sampling period, the control period, as well as the update period for the reinforcement learning. Although we never perform numerical integration for Eq. (16), we notice that Eq. (16) as a time-discretized version of Eq. (12) or Eq. (13) can be rewritten as

19 $$\begin{aligned} \begin{pmatrix} \theta _{n+1} \\ \omega _{n+1} \end{pmatrix}= & {} \begin{pmatrix} 1 &{}\quad \delta t \\ \frac{mgh-k}{I-D\Delta }\delta t &{}\quad 1-\frac{b-P\Delta }{I-D\Delta }\delta t \end{pmatrix}\begin{pmatrix} \theta _{n} \\ \omega _{n} \end{pmatrix}\nonumber \\{} & {} -\begin{pmatrix} 0 \\ \frac{\delta t}{I-D\Delta } \end{pmatrix}\begin{pmatrix} P&\quad D \end{pmatrix}\begin{pmatrix} \theta _{n} \\ \omega _{n} \end{pmatrix}+\begin{pmatrix} 0 \\ \hat{\sigma }\xi _n \end{pmatrix}. \end{aligned}$$

For the system matrix $A_\Delta (P,D)$ of Eq. (13) defined above, denoting the matrix of the first term of Eq. (19) by $\hat{A}_\Delta$, the column vector of $(0\; \; \delta t /(I-D\Delta ))^T$ by $\hat{B}_\Delta$, and the state vector by $x_n=(\theta _n\; \; \omega _n)^T$, we have the following abstract form of the state equation:

20 $$\begin{aligned} x_{n+1}=\hat{A}_\Delta x_n+\hat{B}_\Delta u_n+\hat{\Sigma }\xi _n, \end{aligned}$$

in a form similar to the standard linear feedback control system, by which $K\triangleq (P\; \; D)$ and $u_n\triangleq -Kx_n=-P\theta _n-D\omega _n$ can be viewed as the feedback gain and the feedback control torque, respectively. Moreover, if there is no delay $(\Delta =0)$, all equations defined by Eqs. (12), (13), (19) and (20) become the following simple linear feedback control system:

21 $$\begin{aligned} x_{n+1}=\hat{A}_0 x_n+\hat{B}_0 u_n+\hat{\Sigma }\xi _n, \end{aligned}$$

for which the optimal feedback control gains for the linear quadratic regulator (LQR) may be determined analytically.

### A finite Markov decision process and reinforcement learning

We consider optimal feedback control strategies that can stabilize the upright position of the inverted pendulum model represented by Eq. (18) for several types of cost functions. Specifically, we explore optimal feedback gains *P* and *D* that are allowed to vary depending on the state of the pendulum. Namely, we consider the feedback gain $K\triangleq (P,D)$ as a function of *x*, i.e., $K(x)=(P(x), D(x))$, and explore the optimal distribution of *K*(*x*) over the phase plane of *x* using reinforcement learning (more specifically, using a method of the dynamic programing with a value iteration). Several types of instantaneous cost functions were examined to identify the one that leads to the intermittent control strategy.

Prior to solving such an optimization problem, we discretize the state space of the model as well as the actions characterized by the feedback ankle joint torque, in order to make the problem simple (Suzuki et al. 2023), without use of the neural-network-based function approximators for a value function (critic) and for an action generator (actor). By the discretization as summarized later, Eq. (18) can be considered as a model for a finite Markov decision process. Specifically, we consider a Markov decision process $\mathcal {M}(\pi )$ characterized by $\{\mathcal {X}, \mathcal {A}, p_T, r, \pi \}$, where

- $\mathcal {X}\ni x$ is a set of the finite states
- $\mathcal {A}\ni a$ is a set of the finite actions
- $p_{T}(x_{n+1}| x_n, a):\mathcal {X}\times \mathcal {X}\times \mathcal {A}\rightarrow [0,1]$ is a state transition probability matrix
- $r(x_{n+1}, x_n, a):\mathcal {X}\times \mathcal {X}\times \mathcal {A}\rightarrow \mathbb {R}$ is an instantaneous cost function
- $\pi (x):\mathcal {X} \rightarrow \mathcal {A}$ is a deterministic policy.

Each element of $\mathcal {M}(\pi )$ is defined in this sequel.

#### A set of the finite states $\mathcal {X}$

We consider a rectangular region $\mathcal {D}_{\text {IN}}$ in the *x*-plane ($\theta$–$\omega$ plane) centered at the origin, satisfying $0\le |\theta |\le 0.0505$ and $0\le |\omega |\le 0.0305$, referred to as the state domain. The state domain $\mathcal {D}_{\text {IN}}$ was discretized into square-shaped small elements (6161 elements in total). For each element, the horizontal and vertical side lengths were set to $\delta \theta =1.0\times 10^{-3}$ rad and $\delta \omega =1.0\times 10^{-3}$ rad/s, respectively. The center point of each element was defined as a discrete state, and a set of all such center points was referred to as $\mathcal {X}_{\text {IN}}$. Let $\mathcal {D}_{\text {OUT}}$ be the region outside the $\mathcal {D}_{\text {IN}}$ in the *x*-plane. If a state of the pendulum is located in $\mathcal {D}_{\text {OUT}}$, regardless of its position in $\mathcal {D}_{\text {OUT}}$, such a state is considered as a unique state, referred to as $\mathcal {X}_{\text {OUT}}$ representing a fall. Thus, including the fall-state, we consider a set of 6162 finite states of the inverted pendulum in total, referred to as $\mathcal {X}\triangleq \mathcal {X}_{\text {IN}}\cup \mathcal {X}_{\text {OUT}}$, which is the discretized version of $\mathcal {D}\triangleq \mathcal {D}_{\text {IN}}\cup \mathcal {D}_{\text {OUT}}$.

#### A set of the finite actions $\mathcal {A}$

We consider the active feedback control torque only in the form of the PD controller with the state-dependent gains for the system of Eq. (18). That is, in terms of the Euler–Maruyama version of Eq. (18) described by Eq. (20), we consider the active feedback control torque in the form of

22 $$\begin{aligned} u_n=-K(x_n)x_n=-P(x_n)\theta _n-D(x_n)\omega _n. \end{aligned}$$

Note that values of the *P* and *D* gains affect dynamics of the system with Eq. (18), not only simply through the way of usual PD-feedback control, but also through the system matrix $A_\Delta (P,D)$. Since the gains $P(x_n)$ and $D(x_n)$ that parameterize the matrix $A_\Delta$ are the function of $x_n$, the system of Eq. (18) is a nonlinear control system.

**Fig. 4:**
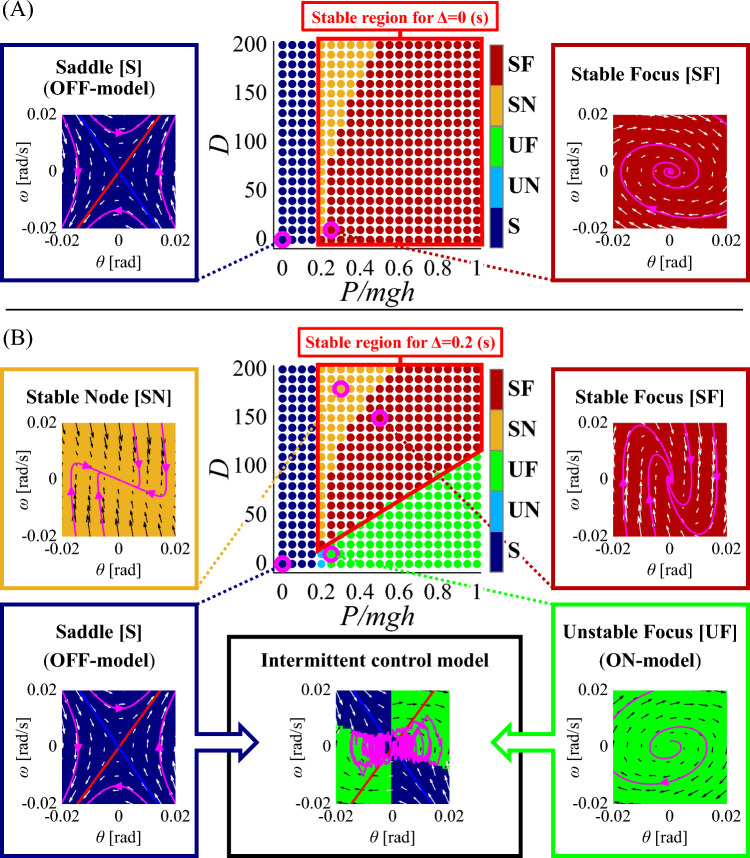
The *P*–*D* domain was discretized into a set of (*P*, *D*)-points that represent the finite set of actions of feedback control. A topological property of the system with each specific action *a* (for a continuous feedback controller) defined by the corresponding (*P*, *D*)-point is color-coded, as shown in the middle panel of each of (**A**) and (**B**), which is referred to as the *topology color map*. The panels in **A** and **B** are for the systems with no feedback delay $(\Delta =0$ s) and with delay $(\Delta =0.2$ s), respectively. Dynamics of the continuous control model with specific (*P*, *D*)-actions are represented by the phase portraits of the model. In **A**, dynamics of the model with two (*P*, *D*)-points, indicated by the pink open circles at $(P,D)=(0,0)$ and $(P,D)=(0.25mgh,10)$, are characterized as a saddle (denoted by S) and a stable focus (denoted by SF), respectively. In **B**, dynamics of the model with two (*P*, *D*)-points, the same points as (**A**) are characterized as a saddle [S] and an unstable focus [UF], respectively. The instability of the model with $(P,D)=(0.25mgh,10)$ is due to the feedback delay. Dynamics of two additional points at $(P,D)=(0.3mgh,180)$ and $(P,D)=(0.5mgh,150)$ are characterized as a stable node (SN) and a stable focus (SF), respectively. The intermittent control model, which switches between two unstable continuous delay feedback control models ($\Delta =0.2$ s) defined by the system with the null action $(P,D)=(0,0)$ and that with the small-gain action $(P, D)=(0.25mgh, 10)$, is characterized by the phase portrait shown in the lower-middle of (**B**), where the phase plane is divided into two regions, $\text {S}_{\text {off}}$ colored by the dark blue and $\text {S}_{\text {on}}$ colored by the green. See text for more details

The range of possible values of $P(x_n)$ and $D(x_n)$ for any state $x_n\in \mathcal {X}$ is set as $0\le P(x_n)\le 1.0mgh$ Nm and $0\le D(x_n)\le 200$ Nms/rad, which defines the *P*–*D* domain. The *P*–*D* domain is discretized into a set of parameter points (*P*, *D*), referred also to as *P*–*D* pairs, by the step sizes of $\delta P=0.05mgh$ Nm and $\delta D=10$ Nms/rad, by which we define a set of 441 (*P*, *D*)-points, referred to as the set of finite actions $\mathcal {A}$. That is, each $(P_i, D_i)$-point represents an action $a_i\in \mathcal {A}$ ($i=1,\ldots ,441$) that will be selected by a policy $\pi$ and used for a state transition from $x_n$ to $x_{n+1}$. Dynamics of the system with a selected action are characterized by the topological property of the equilibrium and a phase portrait of the system by assuming hypothetically that the action selected for the current state $x_n$ is employed persistently and uniformly over the state points $x\in \mathcal {X}$. In other words, we characterize dynamics of the system described by

23 $$\begin{aligned} \frac{dx}{dt}=A_\Delta (P_i,D_i)x, \end{aligned}$$

with the action $a_{i}=(P_i, D_i)$ selected for the current state $x_n$ using the topological property of the equilibrium at $x=0$ for Eq. (23). Figure 4A exemplifies such characterizations of the system in the case with no feedback delay ($\Delta =0$ s). The middle panel of (A) for the *P*–*D* domain is filled by the 441 $(P_i, D_i)$-points for the corresponding actions $a_{i}$ ($i=1,\ldots ,441$). Each $(P_i, D_i)$-point is colored differently, depending on the topological property of the equilibrium of the system of Eq. (23). We call such a colored *P*–*D* domain a *topology color map*. For example, the left-bottom point of the topology color map in Fig. 4A, indicated by a pink open circle for the null action with $(P,D)=(0,0)$, is colored by the dark blue, which means that the equilibrium point of the system with the null action, i.e., when the feedback controller is switched off persistently, is topologically classified as a saddle (denoted by [S]). The phase portrait of the system in this case is depicted by the left panel of Fig. 4A, where the phase plane is also colored by the dark blue that is used to color the (*P*, *D*)-point at (0, 0) in the topology color map. Moreover, in this case, the hyperbolic vector field, the stable manifold (blue line) and the unstable manifold (red line) of the saddle and a few sample trajectories are shown. Another example of (*P*, *D*)-point, located at $(P, D)=(0.25mgh, 10)$ also indicated by another pink open circle in Fig. 4A, is colored by the red, which means that the equilibrium point of the system with the action determined by those small *P*–*D* values is topologically classified as a stable focus (denoted by [SF]). The phase portrait of the system in this case is depicted by the right panel of Fig. 4A, where the phase plane is also colored by the red as is used to color the (*P*, *D*)-point at (0.25*mgh*, 10) in the topology color map. Moreover, the converging focal vector field and a sample of the stable spiral trajectory are shown in the red phase plane. Note that, in the case with $\Delta =0$ s, the system of Eq. (23) is stable if $P_i>(mgh-k)=0.2 mgh$, and the corresponding stability region in the *P*–*D* domain is surrounded by the red thick box in Fig. 4A-middle.

The upper middle panel of Fig. 4B is another topology color map for the system with a feedback delay of $\Delta =0.2$ s. The topology color map in this case is colored differently, compared to Fig. 4A, due to the delay-induced instability of the system. The instability occurs at the lower part of the *P*–*D* domain, as indicated by the green region of the topology color map. The left-bottom point of the topology color map, for the null action with $(P,D)=(0,0)$, is colored by the dark blue as in the case with $\Delta =0$ s, i.e., the equilibrium of the system is a saddle point with the phase portrait shown in the lower-left panel of Fig. 4B that is the same as the left-panel of Fig. 4A. However, the (*P*, *D*)-point at (0.25*mgh*, 10) is now colored by the green, which means that the equilibrium point of the system with the corresponding action of the small *P*–*D* values is topologically classified as an unstable focus (denoted by [UF]). The phase portrait of the system in this case is shown in the lower right panel of Fig. 4B, where the phase plane is green using the same color for the (*P*, *D*)-point at (0.25*mgh*, 10). Moreover, the diverging focal vector field and a sample of unstable spiral trajectory are shown in the green phase plane.

A typical setup of the intermittent control model describe in Fig. 3E and F can be characterized by the actions for two (*P*, *D*)-points on the topology color map and the corresponding phase portraits (Asai et al. 2009). Namely, the intermittent control model that switches between unstable off-subsystem and unstable on-subsystem is defined by the system with the null action $(P,D)=(0,0)$ for the off-subsystem and that with the small-gain action $(P, D)=(0.25mgh, 10)$ for the on-subsystem representing the delay feedback controller with $\Delta =0.2$ s. This situation is illustrated by the phase portrait shown in the lower-middle of Fig. 4B, where the phase plane is divided into two regions, $\text {S}_{\text {off}}$ colored by the dark blue and $\text {S}_{\text {on}}$ colored by the green. Note that, in this case, a sample trajectory of the intermittent control model is generated in the presence of noise ($\sigma =0.2$ Nm). In Fig. 4B, we exemplify dynamics of the system with two more actions, i.e., two (*P*, *D*)-points, using their phase portraits: one for stable node denoted by [SN] with orange color for the (*P*, *D*)-point at (0.3*mgh*, 180), and the other for stable focus denoted by [SF] with red color for (*P*, *D*)-point at (0.5*mgh*, 150). These two parameter sets are closer to the one about (*P*, *D*)-point at (0.5*mgh*, 270) used frequently for the traditional stiffness control model (Peterka 2002; Maurer and Peterka 2005).

#### A state transition probability matrix

A state transition from $x_n$ to $x_{n+1}$ takes place at a time step *n*, according to Eq. (18) with a selected action $a\in \mathcal {A}$ at $x_n$. We consider a deterministic policy $\pi$ as a map from *x* to $a=\pi (x)$, by which a point of (*P*(*x*), *D*(*x*))-pair is selected from $\mathcal {A}$ in the *P*–*D* domain for the discretized state $x\in \mathcal {X}$. Specifically, the state transition probability $p_{T}(x_{n+1}| x_n, a)$ is defined by using a stochastic map $\mathcal {P}:\mathcal {X}\rightarrow \mathcal {X}$ that causes a state transition $x_n\mapsto x_{n+1}$. See “Appendix B” for more details. Briefly speaking, the map $\mathcal {P}$ is defined by a composition of four maps as follows:

24 $$\begin{aligned} \mathcal {P}(x)\triangleq \mathcal {C}\circ \mathcal {N}\circ \mathcal {G}\circ \mathcal {F}(x), \end{aligned}$$

where the first map $\mathcal {F}$ is the deterministic state transition from a discretized state $x_n\in \mathcal {X}$ to a non-discretized state $x_{n+1}^{F}\in \mathcal {D}$ defined as

25 $$\begin{aligned} x^F_{n+1}=\phi \left( x_n,\delta t;P(x_n),D(x_n)\right) \triangleq \mathcal {F}(x_n;a) \end{aligned}$$

by Eq. (15), where $a=\pi (x_n)$ that selects a pair of gains $P(x_n)$ and $D(x_n)$. If $x^F_{n+1}=(\theta ^F_{n+1}\; \; \omega ^F_{n+1})^T\in \mathcal {D}_{\text {IN}}$, the point $x^F_{n+1}$ is mapped to a point $x_{n+1}^{G}$ stochastically, which is represented by the map $\mathcal {G}:\mathcal {D}_{\text {IN}}\rightarrow \mathcal {D}$ as

26 $$\begin{aligned} x^G_{n+1}=\mathcal {G}\left( x^F_{n+1}\right) . \end{aligned}$$

The map $\mathcal {G}$ determines a small random variation of $x^F_{n+1}$ in the $\theta$ direction on the $\theta$–$\omega$ plane. Such stochasticity of $\mathcal {G}$ is not directly associated with the torque noise $\hat{\sigma }\xi _n$, but it is necessary for approximating random dynamics of the second order ODE system with an additive torque noise (Zorzano et al. 1999). We define probability of $p_G(x^G_{n+1})$ as detailed in “Appendix B”. The point $x_{n+1}^{G}$ is further perturbed randomly by the torque noise $\hat{\Sigma }\xi _{n}$, which causes a vertical random displacement in the $\omega$-direction on the $\theta$–$\omega$ plane, according to the Gaussian distribution with the standard deviation of $\hat{\sigma }$. This stochastic mapping $\mathcal {N}: \mathcal {D}_{\text {IN}}\rightarrow \mathcal {D}$ is represented as

27 $$\begin{aligned} x^N_{n+1}=\mathcal {N}\left( x^G_{n+1}\right) . \end{aligned}$$

Finally, the point $x^N_{n+1}$ is mapped to the center of the small element $\Delta \mathcal {D}_{\text {IN}}$ or the fall state $\mathcal {D}_{\text {OUT}}$ that includes $x^N_{n+1}$ by $\mathcal {C}: \mathcal {D}\rightarrow \mathcal {X}$, which is represented by

28 $$\begin{aligned} x_{n+1}=\mathcal {C}\left( x^N_{n+1}\right) . \end{aligned}$$

We define a probability of having $x_{n+1}=\mathcal {C}\circ \mathcal {N}(x^{G}_{n+1})$ denoted by $p_{CN}\left( x_{n+1}=\mathcal {C}\circ \mathcal {N}(x^G_{n+1})\right)$ using the Gauss error function as detailed in “Appendix B”. Note that the point $x_{n+1}\in \mathcal {X}$ could be either in $\mathcal {X}_{\text {IN}}$ or $\mathcal {X}_{\text {OUT}}$, in which the latter means a fall of the pendulum.

In summary, $p_T(x_{n+1}|x_{n},a)$, the probability of the state transition of $x_n \mapsto x_{n+1}$ for a given action *a* characterized by $K(x_{n})=(P(x_n),D(x_n))$ is defined as

29 $$\begin{aligned}{} & {} p_T(x_{n+1}|x_{n},a) \propto p_{CN}\left( x_{n+1}=\mathcal {C}\circ \mathcal {N}(x_{n+1}^{G})\right) \nonumber \\{} & {} \quad \cdot p_G\left( x_{n+1}^{G}=\mathcal {G}\circ \mathcal {F}(x_n;a)\right) \end{aligned}$$

The actual values of the state transition probability are determined by normalizing the probability in Eq. (29) so that the equality

30 $$\begin{aligned} \sum _{x_{n+1}\in \mathcal {X}}p_T(x_{n+1}|x_{n},a)=1 \end{aligned}$$

holds for each action $a\in \mathcal {A}$. In this way, we prepare a set of $|\mathcal {A}|$ state transition probability matrices with the size of $|\mathcal {X}|\times |\mathcal {X}|$, where $|\mathcal {A}|$ and $|\mathcal {X}|$ represent the numbers of element of the sets $\mathcal {A}$ and $\mathcal {X}$, respectively.

#### Instantaneous cost functions

The purpose of this study is to find an instantaneous cost function $r(x_{n+1}, x_n, a)$ that leads to the deterministic policy $\pi (x_n)$ characterizing the intermittent control strategy. We consider two types of simple instantaneous cost functions for a state transition from $x_n$ to $x_{n+1}$ by the action $a=\pi (x_n)$: The first cost function is similar to the one used for the linear quadratic regulator (LQR), referred to as $r^{QR}(x_{n+1}, x_n, \pi (x_n))$, with a state-dependent gains. The other is a modification of $r^{QR}(x_{n+1}, x_n, \pi (x_n))$, referred to as $r^{QRPD}(x_{n+1}, x_n, \pi (x_n))$, which is introduced specifically in this study.

The cost function $r^{QR}$ is defined as follows:

31 $$\begin{aligned}&r^{QR}(x_{n+1}, x_{n}, \pi (x_n))\nonumber \\&={\left\{ \begin{array}{ll} x_n^T Qx_n+u(x_n)^T Ru(x_n) &{}\quad \text {if}\; x_{n+1}\in \mathcal {X}_{\text {IN}} \\ 100 &{}\quad \text {otherwise if}\; x_{n+1}\in \mathcal {X}_{\text {OUT}} \end{array}\right. } \end{aligned}$$

where $u(x_n)=-K(x_n)x_n=-(P(x_n)\; \; D(x_n))x_n$ is the feedback torque at $x_n$ for a selected action $a=\pi (x_n)$ represented by $K(x_n)=(P(x_n)\; \; D(x_n))$. *Q* is a $2\times 2$ semi-definite diagonal matrix, and *R* is a scalar. In this way, if $x_{n+1}$ is not the fall-state, the instantaneous cost, which is determined by the sum of the deviation (or the error) from the upright position and the power consumed by the active feedback torque, will be paid. If $x_{n+1}$ is the fall-state, a large punishment cost will be paid.

The cost function $r^{QRPD}(x_{n+1}, x_n, \pi (x_n))$ is defined as follows:

32 $$\begin{aligned} \begin{array}{ll} &{} r^{QRPD}(x_{n+1}, x_n, \pi (x_n)) = {\left\{ \begin{array}{ll} x_n^T Qx_n+u(x_n)^T Ru(x_n)+w_PP(x_n)&{} \\ \quad +w_DD(x_n) &{}\quad \text {if}\; x_{n+1}\in \mathcal {X}_{\text {IN}} \\ 100 &{}\quad \text {otherwise if}\; x_{n+1}\in \mathcal {X}_{\text {OUT}} \end{array}\right. } \end{array} \end{aligned}$$

where $w_P$ and $w_D$ are the non-negative weight coefficients. This cost is a modification of $r^{QR}$ by adding additional terms of *P*(*x*) and *D*(*x*), by which the reinforcement learning will pursue the small feedback gains, as well as the small error and power. Note that $r^{QR}$ is a special case of $r^{QRPD}$ with $w_P=w_D=0$.

#### The optimal policy determined by the dynamic programing

The optimal deterministic policy $a=\pi (x_n)$ and the associated optimal value function $V^{\pi }(x_n)$ are determined based on the value function defined as

33 $$\begin{aligned} V^{\pi }(x)\triangleq \mathbb {E}_{\pi }\left[ \sum _{k=0}^\infty \gamma ^k r(x_{k+1}, x_k,\pi (x_k)) \Bigg | x_0=x \right] . \end{aligned}$$

where $\mathbb {E}_{\pi }$ represents the expectation conditional on a given Markov decision process $\mathcal {M}(\pi )$, and $\gamma \in [0,1)$ is a discount rate. The optimal value function $V^{\pi }(x)$ is defined by the following Bellman equation as

34 $$\begin{aligned} V^{\pi }(x){} & {} =\min _{a=\pi (x)\in \mathcal {A}}\Bigg \{ \sum _{x'\in \mathcal {X}}p_T(x'|x,\pi (x))\nonumber \\{} & {} \qquad [r(x', x, \pi (x))+\gamma V^{\pi }(x')]\Bigg \} \end{aligned}$$

where $p_T(x'|x,\pi (x))$ is the state transition probability defined above, and the associated optimal deterministic policy is defined by Sutton and Barto (1999)

35 $$\begin{aligned} \pi (x)=\mathop {\textrm{argmin}}\limits _{a\in \mathcal {A}}\Biggl \{ \sum _{x'}p_T(x'|x,a)[r(x', x, a)+\gamma V^{\pi }(x')]\Biggr \}. \end{aligned}$$

In this study, the optimal value function and the optimal deterministic policy are determined using the value iteration algorithm.

#### Exploration of the learning environment leading to the intermittent control policy

In this study, we explore the effect of the motor learning environment, such as the cost function, the discount rate, intensity of the process noise and the feedback time-delay, on the optimal policy and the corresponding sway dynamics of the inverted pendulum, and seek a typical set of the parameters that leads to a control strategy similar to the intermittent control, using the setup of the Markov decision process $\mathcal {M}(\pi )$ defined above. More specifically, we explore the parameter space of $\{Q, R, w_P, w_D, \gamma , \sigma , \Delta \}$ and try to find a typical parameter set for which the optimal deterministic policy $\pi (x)$, i.e., distribution of the action determined by the feedback gains of *P*(*x*) and *D*(*x*) over the state space $x\in \mathcal {X}$, is close to that for the intermittent control strategy (Fig. 4B, lower-middle). Namely, the optimal policy obtained through the reinforcement learning with a variety of parameter sets of $\{Q, R, w_P, w_D, \gamma , \sigma , \Delta \}$ will be compared with the distribution of *P*(*x*) and *D*(*x*) for the typical intermittent controller, denoted by $P_I(x)$ and $D_I(x)$ with

36 $$\begin{aligned} \begin{array}{llll} P_{I}(x)=0.25mgh &{} \text{ and } &{} D_{I}(x)=10 &{} \text{ for } x\in \text {S}_{\text {on}} \\ P_{I}(x)=0 &{} \text{ and } &{} D_{I}(x)=0 &{} \text{ for } x\in \text {S}_{\text {off}}. \end{array} \end{aligned}$$

## Results and discussion

**Fig. 5:**
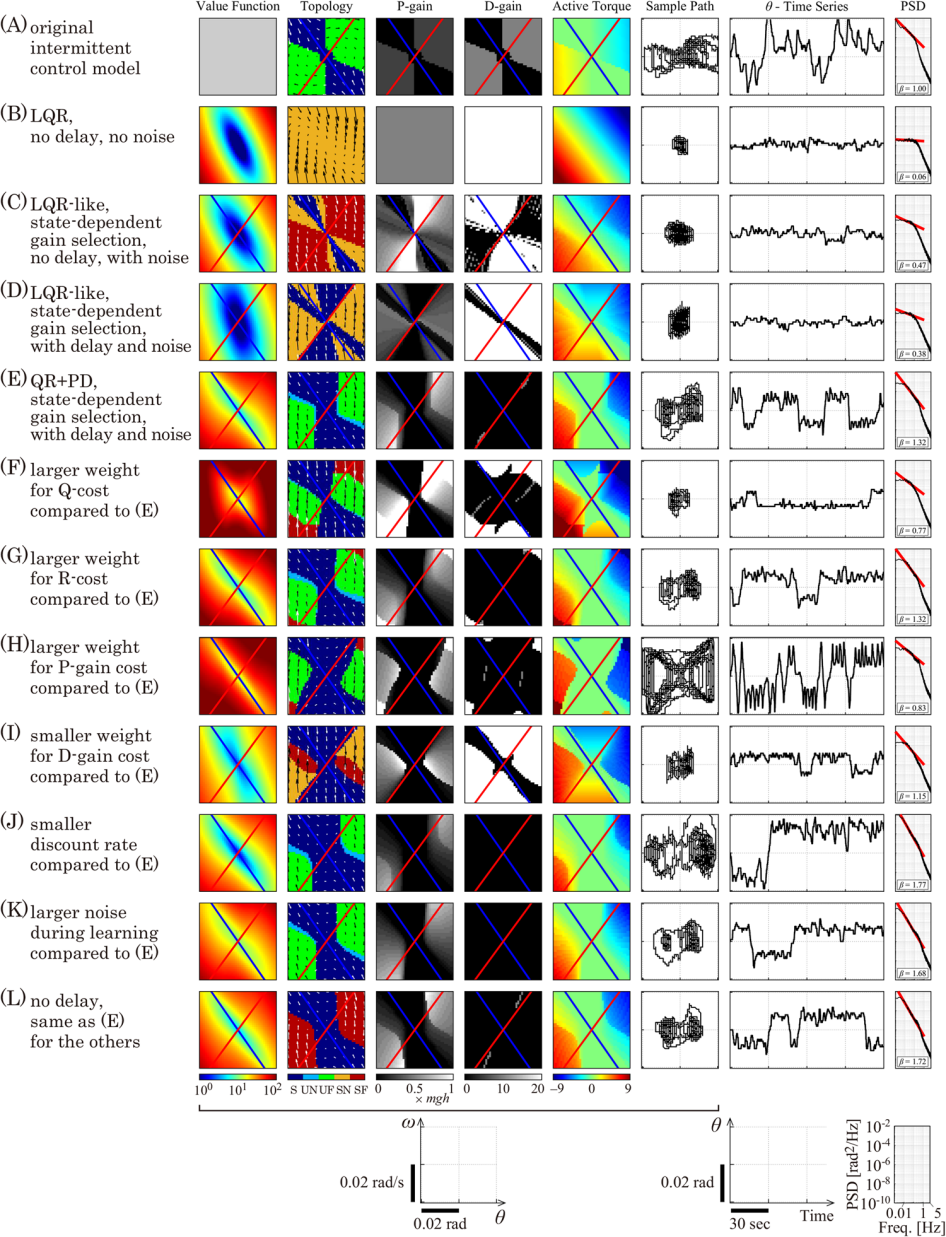
Control policy and dynamics of the model with various optimal controllers obtained for various sets of $\{Q, R, w_P, w_D, \gamma , \sigma , \Delta \}$. For each row, from the left to the right, the optimal value function $V^{\pi }(x)$, the topology color map, state-dependent policy for selecting *P*-gain, state-dependent policy for selecting *D*-gain, state-dependent active torque, a sample path of the stochastic dynamics, the corresponding time series of $\theta$, and the PSD of the time series of $\theta$. Deterministic dynamics of the model controlled by acquired policy are characterized by the topological property of the equilibrium, including saddle [S] (dark blue), unstable node [UN] (blue), unstable focus [UF] (green), stable node [SN] (orange), and stable focus [SF] (red). Values of $(Q, R, w_P, w_D, \gamma , \sigma , \Delta )$: **A** The intermittent control model ($\sigma =0.2$, $\Delta =0.2$). **B** $(500I_2, 0.025, 0, 0, 1.0, 0, 0)$, **C** $(500I_2, 0.025, 0, 0, 0.99, 0.2, 0)$, **D** $(500I_2, 0.025, 0, 0, 0.99, 0.2, 0.2)$. **E** $(500I_2,0.025,0.001,0.05, 0.99, 0.2, 0.2)$, **F** $(5000I_2,0.025,0.001,0.05, 0.99, 0.2, 0.2)$, **G** $(500I_2,0.05,0.001,0.05, 0.99, 0.2, 0.2)$, **H** $(500I_2,0.025,0.005,0.05, 0.99, 0.2, 0.2)$, **I** $(500I_2,0.025,0.001,0.001, 0.99, 0.2, 0.2)$, **J** $(500I_2,0.025,0.001,0.05, 0.985, 0.2, 0.2)$, **K** $(500I_2,0.025,0.001,0.05, 0.99, 0.4, 0.2)$, **L** $(500I_2,0.025,0.001,0.05, 0.99, 0.2, 0)$. See text for details

Results of our exploration of the instantaneous cost function in the parameter space of $\{Q, R, w_P, w_D, \gamma , \sigma , \Delta \}$ for Eq. (32) are summarized in Fig. 5. Control policy and dynamics of the model with various optimal controllers obtained for various sets of $\{Q, R, w_P, w_D, \gamma , \sigma , \Delta \}$ are compared with the control policy and dynamics of the intermittent control model.

The control policy defined by $P_I(x)$ and $D_I(x)$ in Eq. (36) and dynamics of the intermittent control model for those comparisons are depicted in Fig. 5A, i.e., the top row of Fig. 5, in which the topology color map (topology panel), state-dependent policy for selecting *P*-gain on the $\theta$–$\omega$ plane (*P*-gain panel), state-dependent policy for selecting *D*-gain on the $\theta$–$\omega$ plane (*D*-gain panel), state-dependent active torque (active torque panel), a sample path of the stochastic dynamics (sample path panel), the corresponding time series of $\theta$ (time series panel), and the PSD of the time series of $\theta$ (PSD panel) are shown in the 2nd to 8th column of Fig. 5A, respectively. Note that each PSD shown in the 8-th column of Fig. 5 was obtained by an ensemble average of 30 PSDs that were computed by FFT of statistically independent 30 $\theta$ time series of length 1310.72 s. The first column of Fig. 5 is reserved for presenting a value function of the model with a given instantaneous cost function (value function panel), which is absent for Fig. 5A. The topology panel of Fig. 5A is exactly the same as the lower middle panel of Fig. 4B. In the *P*-gain panel of Fig. 5A, the off-region and the on-region of the $\theta$–$\omega$ plane are colored by black ($P(x)=0$ for $x\in \text {S}_{\text {off}}$) and dark gray (small gain with $P(x)=0.25mgh$ for $x\in \text {S}_{\text {on}}$), respectively. Similarly, in the *D*-gain panel of Fig. 5A, the off-region and the on-region of the $\theta$–$\omega$ plane are colored by black ($D(x)=0$ for $x\in \text {S}_{\text {off}}$) and gray (small gain with $D=10$ for $x\in \text {S}_{\text {on}}$), respectively. For those panels, the stable manifold of the saddle point (blue line) crosses the center of the off-region. The active torque shown in the active torque panel of Fig. 5A is null for the off-region that is colored by green, whereas it exhibits negative (blue) and positive (yellow) values in the right-upper and left-lower on-regions of the $\theta$–$\omega$ plane. The trajectory of the stochastic dynamics in the sample path panel of Fig. 5A is butterfly-wing-shaped that characterizes the intermittent control strategy. PSD of postural sway exhibit a power-law-like scaling in the low frequency band ($\beta \sim 1.0$).

### LQR-like cost functions: cases with $w_P=w_D=0$

Control policy and dynamics of the model with no delay ($\Delta =0$) and with the optimal controller for the LQR cost function that uses $r^{QR}$ in Eq. (31) or $r^{QRPD}$ with $w_P=w_D=0$ in Eq. (32) with $Q=500I_2$, $R=0.025$ and $\gamma =1$ are shown in Fig. 5B, where $I_2$ is the $2\times 2$ identity matrix. Note that the weighting coefficients $Q=500I_2$ and $R=0.025$ are used as the reference for the comparison in this sequel, which were determined as a result of trial and error. In this simple LQR case, we consider the state-independent optimal *P* and *D* gains as assumed usually for LQR problem, and they are computed for the state equation of Eq. (21) for $\Delta =0$ using the discrete-time algebraic Riccati equation under noiseless deterministic situation ($\sigma =0$), which leads to $P(x)=0.398mgh$ Nm/rad and $D(x)=163$ Nms/rad, regardless of the state *x*. In this way, the *P*-gain panel and the *D*-gain panel are colored in Fig. 5B, respectively, by gray (for medium value of *P*, compared to the *P* value for the intermittent control model) and by white (for very large value of *D*, compared to the *D* value for the intermittent control model) uniformly over the $\theta$–$\omega$ plane. The value function in this case is a quadratic surface (value function panel in Fig. 5B). The equilibrium point is classified as the stable node [SN], by which the $\theta$–$\omega$ plane is colored by orange (topology panel in Fig. 5B). The active torque exhibits a linear dependency on $\theta$ and $\omega$, providing the linear gradient of the color on the $\theta$–$\omega$ plane (active torque panel in Fig. 5B). Stochastic dynamics in the sample path panel and the time profile in the time series panel are much less fluctuated compared to the intermittent control model and rigidly clustered around the origin in the presence of the noise with the common intensity ($\sigma =0.2$). PSD of postural sway is with a typical shape for the non-resonant second order system with plateau power in the low frequency band ($\beta \sim 0.06$).

Figure 5C still considers a case with $\Delta =0$ and $r^{QR}$ in Eq. (31) or $r^{QRPD}$ with $w_P=w_D=0$ in Eq. (32) with $Q=500I_2$ and $R=0.025$, as in the LQR case in Fig. 5B, but the *P* and *D* gains are allowed to vary depending on the state *x*. Moreover, the discount rate and the noise intensity are set to $\gamma =0.99$ and $\sigma =0.2$, respectively. Optimal control policy in this case should be obtained numerically using the value iteration method. The most notable difference between Fig. 5B and C appears in the control policy $\pi$ for the state-dependent selection of *P* and *D* values (*P*-gain and *D*-gain panels). In particular, a region on the $\theta$–$\omega$ plane with very small *P* gains (zero gain practically), which is colored by black in the *P*-gain panel of Fig. 5C appears, which can be regarded as the off-region, around the stable manifold of the off-subsystem (the blue line), although the off-region in Fig. 5C is much narrower than the off-region for the intermittent control model in Fig. 5A. The off-region in Fig. 5C for the topology panel is colored by dark blue, indicating topological classification of saddle [S] as in the off-region for the intermittent control model. On the other hand, the *P*-gain around the red line, representing the unstable manifold of the off-subsystem in the *P*-gain panel, exhibits large values as indicated by the white and light gray color regions, in which the topological property of the model becomes stable focus [SF] as indicated by the red regions in the topology panel. The *D*-gain panel, which is uniformly white in Fig. 5B, shows black regions with very small *D*-gain near the 1st and 3rd quadrants. However, configuration of those off-regions is different from that of the intermittent control model.

In Fig. 5D, the feedback delay time $\Delta =0.2$ s is introduced for the parameter environment of Fig. 5C, while keeping the cost-function-related environment ($r^{QR}$ in Eq. (31) or $r^{QRPD}$ with $w_P=w_D=0$ in Eq. (32) with $Q=500I_2$ and $R=0.025$) unchanged, by which the effect of delay $\Delta$ alone on the control policy can be manifested. Off-regions of *P*-gain in Fig. 5D appear on both sides of the stable manifold of the off-subsystem (the blue line), which are broader than those in Fig. 5C. On the other hand, off-regions of *D*-gain in Fig. 5D are narrower than those in Fig. 5C, located near the stable manifold of the off-subsystem. Linear dependency (linear gradient of the color) of the active torque on the $\theta$ and $\omega$, which are present in Fig. 5B and C, tends to be lost.

In summary, the control policy tends to show on–off switching by allowing the *P* and *D* gain to vary as a function of the state *x*, under the LQR-like form of the instantaneous cost function $r^{QR}$ defined by Eq. (31). The off-region appears near the stable manifold of the off-subsystem, implying that use of the vector field near the stable manifold, which directs the saddle point at the origin, is beneficial for reducing both the error (postural deviation from the upright position) and the energy consumption by the active feedback controller. However, size of the off-region selected under the LQR-like form of the instantaneous cost function is narrower than the intermittent control model, although presence of the feedback delay $\Delta$ makes the off-region slightly larger. Moreover, the on-region tends to be accompanied by larger *P*–*D* gains in comparison with the intermittent control model, which makes the controller for the on-region stable, either stable node [SN] or stable focus [SF]. Although appearance of selection of the optimal controllers utilizing switching between unstable off-subsystem and stable on-subsystem is interesting property, these are not necessarily similar to the intermittent controller shown in Fig. 5A.

### LQR-like cost plus feedback gain cost

The reinforcement learning based models (RL-models) with the feedback delay $\Delta$ and the optimal controller under the instantaneous cost function accompanied with the same weights of $Q=500I_2$ and $R=0.025$ as in the LQR case of Fig. 5C and D, but now with $r^{QRPD}$ in Eq. (32) for $w_P=0.001$ and $w_D=0.05$ exhibit dynamics that are one of the most similar to those of the intermittent control model among the examined learning environments, as shown in Fig. 5E. Specifically, configuration of the off-region in the panels of topology, *P*-gain, *D*-gain, and active torque in Fig. 5E are quite similar to those of the intermittent control model in Fig. 5A. Trajectory in the sample path panel in Fig. 5E is with butterfly-wing-shaped as in the interment control model, leading to the PSD with a power-law-like scaling behavior in the low frequency band ($\beta \sim 1.32$). Remarkably, the topological property of the on-regions in this case are unstable focus [UF], by which the on-regions in the topology panel are mostly colored by green, as in the intermittent control model. This is achieved not only by the relatively small *P*-gains in the on-regions, but also by the very small values (practically zero) of the *D*-gains that lead to mostly black colored *D*-gain panel. Moreover, slightly negative slope of the on–off boundary that makes the on-regions protruded from the 1st and 3rd quadrants emphasizes the similarity of the off-region geometry, in comparison with the intermittent control model with the slope of the on–off boundary line determined by the $\omega _\Delta =l\theta _\Delta$, as described in Fig. 3.

There are differences between the RL-model in Fig. 5E and the intermittent control model. Despite the overall similarity of the off-region geometry between two models, the on-regions occupying the 1st and 3rd quadrants are separated by a vertical band for the RL-model, while those are separated only by the circular deadzone around the origin for the intermittent control model. In this sense, the off-regions of the RL-model shown in Fig. 5E combine features of the intermittent control model and the threshold control model shown in Fig. 3. The *P*-gain for the on-region of the RL-model, which is colored by gray in the *P*-gain panel, is slightly larger than the those of the intermittent control model, which is colored by dark gray. On the other hand, the *D*-gain of the RL-model is mostly zero, by which the *D*-gain panel is colored mostly by black, meaning that the *D* gains of the RL-model are smaller than those of the intermittent control model for its on-region. Because of the slightly large *P* gains in the on-regions for the RL-model, active torques in the on-regions for the RL-model are slightly larger than those for the intermittent control model.

Figure 5F characterizes another RL-model with 10 times larger value of *Q*, i.e., showing the effect of the change only in the *Q* value from $Q=500I_2$ used for the RL-model in Fig. 5E to $Q=5000I_2$. The large value of *Q* penalizes the cost more for the postural deviation (error) from the upright position, which makes the cumulative cost larger for this model, compared to the one in Fig. 5E, as shown in the value function panel. Although the off-region geometry for the *P* gain does not change a lot, *P*-gains for the on-region increase. Moreover, upper right and lower left regions of the *D*-gain panel become white, meaning that *D*-gains in those on-regions increase. As a result, the upper right and lower left regions of the topology panel change from green to red, meaning that the model for those on-regions are stable focus [SF]. The large gains in the on-regions makes the fluctuation of the RL-model small, leading to the small scaling exponent in the low frequency band of the PSD ($\beta \sim 0.77$).

Figure 5G characterizes the RL-model with 2 times larger value of *R*, i.e., showing the effect of the change only in the *R* value from $R=0.025$ used for the RL-model in Fig. 5E to $R=0.05$. The large value of *R* penalizes the cost more for the energy consumption by the active torque in comparison with the RL-model in Fig. 5E. In this case, *P* and *D* gains increase at the upper right and lower left corners of the *P* and *D* gain panels, which makes the model for those regions stable focus [SF]. However, the effect on the overall dynamics of the RL-model is limited.

Figure 5H characterizes the RL-model with 5 times larger value of $w_P$, i.e., showing the effect of the change only in the $w_P$ value from $w_P=0.001$ used for the RL-model in Fig. 5E to $w_P=0.005$. The large value of $w_P$ penalizes the cost more for the *P* gain in comparison with the RL-model in Fig. 5E. Against the intuition that expects the RL-model with smaller *P* values, *P* values in the on-region do not decrease apparently. Instead, as a natural consequence of the large penalty for the large *P* gain values, the RL-model exhibits a larger off-region that covers not only the stable manifold, but also the unstable manifold of the off-subsystem. The larger off-region makes the postural fluctuation larger and more clearly oscillatory, compared to that in Fig. 5E. Scaling exponent of the PSD for such oscillatory dynamics in the low frequency band is less than that for Fig. 5E ($\beta \sim 0.83$).

Figure 5I characterizes the RL-model with 1/50 smaller value of $w_D$, i.e., showing the effect of the change only in the $w_D$ value from $w_D=0.05$ used for the RL-model in Fig. 5E to $w_D=0.001$. The small value of $w_D$ does not consider the cost of *D*-gain seriously, in comparison with the RL-model in Fig. 5E, leading to the large increase in *D* values for a wide range of the *D*-gain panel. In this way, the off-region for the *D*-gain panel becomes smaller. Because of the larger on-regions with large *D* gains, the model for those on-regions are stable focus [SF], which makes the postural fluctuation in one of the butterfly-wing smaller, compared to that in Fig. 5E.

Figure 5J characterizes the RL-model with slightly smaller value of the discount rate $\gamma$, i.e., showing the effect of the change only in the $\gamma$ value from $\gamma =0.99$ used for the RL-model in Fig. 5E to $\gamma =0.985$. The small value of $\gamma$ makes the cost less accumulated, meaning that the policy selection tends to be performed less globally. This case, together with next case, provides the RL-models exhibiting dynamics that are similar to those of the intermittent control model, as much as the RL-model with the learning environments used for Fig. 5E. Although the off-region is much broader than the one for the one for Fig. 5E and the one for the intermittent control model, *P*-gains in the on-regions are smaller (colored by darker gray) than the those for the RL-model in Fig. 5E, which makes the *P*-gains closer to the one for the intermittent control model. Note that, in this case, the noise intensity for simulating dynamics of the RL-model was set to $\sigma =0.4$, although the noise intensity for performing the value iteration was set to $\sigma =0.2$. This is because $\sigma =0.2$ for simulating dynamics of the RL-model was too small to induce transitions between the left and the right wings of the butterfly.

Figure 5K characterizes the RL-model with 2 times large value of the noise intensity $\sigma$, i.e., showing the effect of the change only in the $\sigma$ value from $\sigma =0.2$ used for the RL-model in Fig. 5E to $\sigma =0.4$. The large value of $\sigma$ makes dynamics of the pendulum more unpredictable, meaning that the policy selection should be performed with larger uncertainty. As stated above, this case provides the RL-models exhibiting dynamics that are similar to those of the intermittent control model, as much as the RL-model with the learning environments used for Fig. 5E and J. As in the case with Fig. 5J, *P*-gains in the on-regions are smaller (colored by darker gray) than the those for the RL-model in Fig. 5E, which makes the *P*-gains closer to the one for the intermittent control model. Scaling exponent of the PSD in the low frequency band for this case is larger than that for Fig. 5E ($\beta \sim 1.68$). This result suggest that the process noise plays a critical role for making the control policy intermittent using the unstable on-subsystem with small feedback gains. Intuitively, one may expect a selection of large feedback gains under the noisy environment with larger uncertainty in order to make the standing posture rigid. Against such an intuition, the reinforcement learning with noisy environment for the current problem leads to the control policy that selects smaller *P* and *D* gains with the intermittent control. This might be because the intermittent control that exploits the stable manifold of the off-subsystem is much more robust than the threshold control (see Fig. 3) in the presence of feedback delay, and also it is more energetically efficient compared to the continuous control.

Figure 5L examines the effect of the feedback delay $\Delta$ by changing the value of $\Delta$ from $\Delta =0.2$ used for the RL-model in Fig. 5E to $\Delta =0$. It is apparent by the comparison between Fig. 5E and L that dynamics in the on-region for the case with no delay becomes stable focus [SF] with a selection of the large *P*-gains (the red on-regions), while keep the on–off regions geometry mostly unchanged. This result implies that the use of the delay-induced unstable oscillation of the on-subsystem with small *P* gains in the intermittent control model can be replaced by the stable oscillation of the subsystem with the large *P*-gains, which is made possible by the similarity of the vector fields for stable and unstable focus. However, the use of small *P*-gains is much more energetically efficient, which is available by the presence of feedback delay, leading to the selection of the unstable oscillation with small *P*-gains in the reinforcement learning for stabilizing the upright posture.

### Summary and further discussion

In summary, our parameter exploration showed the existence of learning parameters that lead to the intermittent control policy. In particular, a reinforcement learning policy with a balanced tradeoff between error and power, a balance between *P* and *D* gains, and appropriate amounts of the delay and the process noise lead to the intermittent control strategy. Surprisingly, it appears that process noise and feedback delay, instead of being noxious for stability, are crucial for achieving intermittent control that assures a robust form of dynamic stability, alternating between two unstable subsystems. Those properties exploiting instability for stability are in stark contrast to another type of intermittent controller that utilizes a model-based prediction (Gawthrop et al. 2011).

Stochastic dynamics of the RL-models exhibit robust stability, particularly in the case with the models switching between unstable off-subsystem and unstable on-subsystem with small *P* and *D* gains. The robustness is ensured by the geometry of the off-region in the $\theta$–$\omega$ plane. Namely, the geometry of the off-region for the RL-models shown particularly in Fig. 5E, J and K is a kind of combination of the off-region of the intermittent control model shown in Fig. 3D or E and that of the threshold control model shown in Fig. 3B. Although the threshold control model shown in Fig. 3B with the tilt-angle-based threshold mechanism is unstable, the off-region extended into the 2nd and 4th quadrants of the $\theta$–$\omega$ plane, as in the intermittent control model shown in Fig. 3D or E and in the RL-models shown in Fig. 5E, J and K, makes the RL-models stable in a quite robust manner. In those models, the stable eigenmode of the unstable off-subsystem is responsible for the robust stability, in which dynamics of the stable eigenmode of the off-subsystem brings any state point moved into the off-region from the on-region close to the upright position in a robust manner (Asai et al. 2009).

One may wonder why the additional terms of cost for the *P* and *D* gains are required to obtain the intermittent control model. That is, those terms seem redundant, because penalizing values of *P* and *D* gains might be included equivalently in the penalty for the power consumed by the PD feedback controller. However, the feedback control torque represents the sum of proportional and derivative control torques, in which those two types of torque cannot be evaluated separately. On the other hand, the on-subsystem behaving as an unstable focus [UF] in the intermittent control model should be operated not only with a small *P*-gain but also with a small *D*-gain simultaneously. It is difficult for the instantaneous cost function $r^{QR}$ without the cost terms for *P* and *D* gains to lead to RL-models with small *D* gains in a wide range of the $\theta$–$\omega$ plane as is often the case with Fig. 5C and D. Moreover, reduced penalization for *P*-gain can easily result in the increase of *D*-gain in a broad region of the $\theta$–$\omega$ plane as in Fig. 5I. This is why a balanced penalty on *P* and *D* gains separately is required for obtaining the intermittent control policy that alternates between two unstable subsystems.

### Limitations

We used the ODE approximation for the DDE on-subsystem in this study. Although it has been confirmed that the ODE approximation of the original DDE works satisfactory for the current control system (Stepan and Kollar 2000), even for stochastic dynamics in the presence of process noise (Suzuki et al. 2023), it is necessary to examine whether the same conclusion obtained for the ODE approximated on-subsystem can be reached even if we use the DDE on-subsystem, which can be done using methodologies of reinforcement learning developed for systems with delayed actions (Nath et al. 2021). Moreover, the results shown in Fig. 5 compared among only limited sets of the learning parameters, including weighting coefficients in the cost function, noise intensity and discount rate. More intensive parameter explorations are required to validate our conclusion.

We considered a finite state Markov chain by discretizing the state space of the model as well as the actions characterized by the feedback ankle joint torque. Although this simplification allowed us to perform the dynamic programing based evaluations of the optimal controllers, the results of the current paradigm should be examined using neural-network-based function approximators for a value function (critic) and for an action generator (actor), when we consider the system with the DDE on-subsystem.

## Conclusion

The intermittent control model describes a novel strategy for stabilizing human quiet stance (Bottaro et al. 2008; Asai et al. 2009), which is consistent with recent findings of non-spring-like behavior of calf muscles during quiet stance (Loram et al. 2005). It is a hybrid dynamical system that switches between two unstable subsystems, in which a sequence of actions performed by a time-delay proportional (*P*) and derivative (*D*) feedback controller is switched between on and off in a state-dependent manner. In this study, a stochastic delay differential equation, described by the equation of motion of a single inverted pendulum stabilized by a delay PD feedback controller, was approximated by a discrete-time finite state Markov chain. We then considered a state-dependent selection of an action that is performed as a state-dependent selection of a pair of (*P*, *D*) gains. We examined the optimal control policy and associated stochastic dynamics of the pendulum for a Markov decision process that is defined with an instantaneous cost function, represented by a weighted sum of the erroneous deviation from the upright position, the power consumption by the active controller, and the magnitudes of *P*-gain and *D*-gain, in the presence of process noise and feedback delay. We explored parameters for the learning environment, including the weighting coefficients, the discount rate for the cumulative cost, the noise intensity and the delay, and showed that there exist several sets of the learning parameters that lead to the intermittent control policy that switches between unstable on- and off-subsystems. The instantaneous cost function for the reinforcement learning that leads to the intermittent control policy is characterized as follows: It exhibits a tolerance for the displacement from the upright position. It emphasizes reducing the power consumed by the action. It prefers small values of the *D*-gain, which can be achieved by putting weight on the *D*-gain. Appropriate amounts of the process noise and the feedback delay, which are often considered as the source of instability, contribute to reduce the *P*-gains, leading to the intermittent control with switching between two unstable subsystems, which assures a robust form of dynamic stability.

## Figures and Tables

**Fig. 6:**
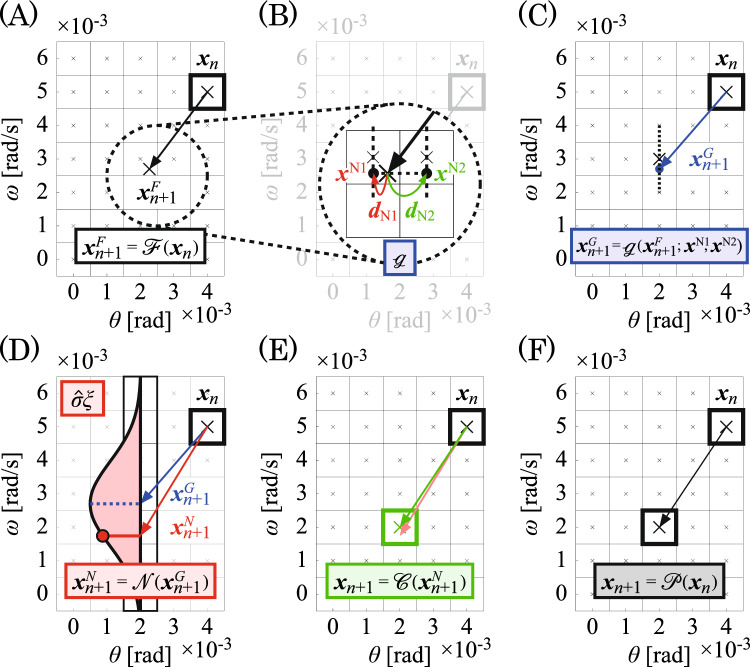
A schematic illustration of how the state transition and its probability of the Markov process model are constructed

## Data Availability

No datasets were generated or analysed during the current study.

## Body parameters in the model

Typical parameter values of the bodily pendulum are summarized in Table 1.

**Table 1:** Values of the body parameters

*I*	60 kg m$^{2}$
*m*	60 kg
*g*	9.81 m/s$^{2}$
*h*	1.0 m
*k*	471 Nm/rad
*b*	4.0 Nms/rad
$\Delta$	0.2 s

## The state transition probability

The state transition probability $p_{T}(x_{n+1}| x_n, a)$ is defined as follows (see Fig. 6). First, the deterministic state transition from a discretized state $x_n$ to a non-discretized state $x_{n+1}^{F}\in \mathcal {D}$ is denoted by the map $\mathcal {F}:\mathcal {X}\rightarrow \mathcal {D}$ defined as

B1 $$\begin{aligned} x^F_{n+1}=\phi \left( x_n,\delta t;P(x_n),D(x_n)\right) \triangleq \mathcal {F}(x_n), \end{aligned}$$

where $x^F_{n+1}$ is not an element of the finite states $\mathcal {X}$, but a point of $\mathcal {D}$. If $x^F_{n+1}=(\theta ^F_{n+1}\; \; \omega ^F_{n+1})^T\in \mathcal {D}_{\text {IN}}$, the point $x^F_{n+1}$ is mapped to a point $x_{n+1}^{G}$ stochastically (Fig. 6A), which is represented by the map $\mathcal {G}:\mathcal {D}_{\text {IN}}\rightarrow \mathcal {D}$ as

B2 $$\begin{aligned} x^G_{n+1}=\mathcal {G}\left( x^F_{n+1}\right) . \end{aligned}$$

The point $x_{n+1}^{G}$ is either $x^\text {N1}$ or $x^\text {N2}$, one of which is selected stochastically. The point $x^\text {N1}$ is located in the small squared element $\Delta \mathcal {D}_{\text {IN}}^\text {N1}$ that includes the point $x^F_{n+1}$. The point $x^\text {N2}$ is in the small squared element, either $\Delta \mathcal {D}_{\text {IN}}^\text {N2}\in \mathcal {D}_{\text {IN}}$ or $\Delta \mathcal {D}_{\text {OUT}}^\text {N2}\in \mathcal {D}_{\text {OUT}}$, located either right-hand or left-hand neighbor of $\Delta \mathcal {D}_{\text {IN}}^\text {N1}$. The pendulum falls, if $x^\text {N2}\in \mathcal {D}_{\text {OUT}}$. We consider the case of $x^\text {N2}\in \Delta \mathcal {D}_{\text {IN}}$ in this sequel. Note that the center point of any small squared element $\Delta \mathcal {D}_{\text {IN}}$ is an element of the discretized finite states $\mathcal {X}$, with the horizontal and vertical sides of the square being $\delta \theta$ and $\delta \omega$ respectively. The point $x^\text {N1}$ is located at the intersection of the vertical line passing through the center of the element $\Delta \mathcal {D}_{\text {IN}}^\text {N1}$ and the horizontal line passing through the point $x^F_{n+1}$. Similarly, the point $x^\text {N2}$ is located at the intersection of the vertical line passing through the center of the element $\Delta \mathcal {D}_{\text {IN}}^\text {N2}$ and the horizontal line passing through the point $x^F_{n+1}$. Probabilities of the stochastic selection of $x^\text {N1}$ and $x^\text {N2}$ as the point $x_{n+1}^{G}$ are determined using the distance between $x^F_{n+1}$ and each of $x^\text {N1}$ and $x^\text {N2}$, respectively, defined as

B3 $$\begin{aligned} d_{\text {N1}}\left( x^F_{n+1}\right)&\triangleq \left| \theta ^\text {N1}\left( x^F_{n+1}\right) -\theta ^F_{n+1}\right| , \end{aligned}$$

B4 $$\begin{aligned} d_{\text {N2}}\left( x^F_{n+1}\right)&\triangleq \left| \theta ^\text {N2}\left( x^F_{n+1}\right) -\theta ^F_{n+1}\right| , \end{aligned}$$

where $\theta ^\text {N1}(x^F_{n+1})$ and $\theta ^\text {N2}(x^F_{n+1})$ are the theta-coordinate values of $x^\text {N1}(x^F_{n+1})$ and $x^\text {N2}(x^F_{n+1})$, respectively. Moreover, $\theta ^F_{n+1}$ is the theta-coordinate value of $x^F_{n+1}$. Since the element $\Delta \mathcal {D}_{\text {IN}}^\text {N1}$ includes the point $x^F_{n+1}$, $x^\text {N1}$ is closer to $x^F_{n+1}$, compared to $x^\text {N2}$. Thus, the inequality of $d_{\text {N1}}(x^F_{n+1})<d_{\text {N2}}(x^F_{n+1})$ holds. With those distances, $x^F_{n+1}$ is mapped to $x^\text {N1}$ with a probability of

B5 $$\begin{aligned}{} & {} p_G\left( x^G_{n+1}=x^\text {N1}\right) \triangleq d_{\text {N1}}\left( x^F_{n+1}\right) /\left( d_{\text {N1}}+d_{\text {N2}}\right) \nonumber \\{} & {} \qquad \qquad \qquad \qquad \qquad \quad =d_{\text {N1}}\left( x^F_{n+1}\right) /{\delta \theta }, \end{aligned}$$

and it is mapped to $x^\text {N2}$ with a complementary probability of

B6 $$\begin{aligned} p_G\left( x^G_{n+1}=x^\text {N2}\right) \triangleq d_{\text {N2}}\left( x^F_{n+1}\right) /{\delta \theta }. \end{aligned}$$

See Fig. 6A and B.

The point $x_{n+1}^{G}$ illustrated in Fig. 6C is perturbed randomly by the additive Gaussian noise $\hat{\Sigma }\xi _{n}$ as the motor noise, which causes vertical displacement in the $\omega$-direction, according to the Gaussian distribution with its mean located at $\omega ^F_{n+1}$ ($\omega$-coordinate value of $x^F_{n+1}$) and the standard deviation of $\hat{\sigma }$. This stochastic mapping $\mathcal {N}: \mathcal {D}_{\text {IN}}\rightarrow \mathcal {D}$ is represented as

B7 $$\begin{aligned} x^N_{n+1}=\mathcal {N}\left( x^G_{n+1}\right) . \end{aligned}$$

Finally, the point $x^N_{n+1}$ is mapped to the center of the small element $\Delta \mathcal {D}_{\text {IN}}$ or the fall state $\mathcal {D}_{\text {OUT}}$ that includes $x^N_{n+1}$ by $\mathcal {C}: \mathcal {D}\rightarrow \mathcal {X}$, which is represented as

B8 $$\begin{aligned} x_{n+1}=\mathcal {C}\left( x^N_{n+1}\right) . \end{aligned}$$

By definition of $\mathcal {N}$, the probability of having $x_{n+1}=\mathcal {C}\circ \mathcal {N}(x^{G}_{n+1})$ is given by

B9 $$\begin{aligned}{} & {} p_{CN}\left( x_{n+1}=\mathcal {C}\circ \mathcal {N}\left( x^G_{n+1}\right) \right) \nonumber \\{} & {} \quad =\text {erf}\left( \frac{\text {upper}\left[ x^G_{n+1}\right] -x^G_{n+1}}{\sqrt{2}\hat{\sigma }}\right) \nonumber \\{} & {} \qquad -\text {erf}\left( \frac{\text {lower}\left[ x^G_{n+1}\right] -x^G_{n+1}}{\sqrt{2}\hat{\sigma }}\right) , \end{aligned}$$

where erf is the Gauss error function, and upper$[x^G_{n+1}]$ and lower$[x^G_{n+1}]$ represent, respectively, the $\omega$-coordinate values of the upper and the lower sides of the small-squared element $\Delta \mathcal {D}_{\text {IN}}$ that includes the point $x^G_{n+1}$. The point $x_{n+1}\in \mathcal {X}$ could be either in $\mathcal {X}_{\text {IN}}$ or $\mathcal {X}_{\text {OUT}}$. In this way, stochastic state transition $x_n\mapsto x_{n+1}$ by one time step is defined by $\mathcal {P}:\mathcal {X}\rightarrow \mathcal {X}$ as

B10 $$\begin{aligned} x_{n+1}=\mathcal {C}\circ \mathcal {N}\circ \mathcal {G}\circ \mathcal {F}(x_n)\triangleq \mathcal {P}(x_n). \end{aligned}$$

In summary, $p_T(x_{n+1}|x_{n},a)$, the probability of the state transition of $x_n \mapsto x_{n+1}$ for a given action *a* characterized by $K(x_{n})=(P(x_n),D(x_n))$ is defined as

B11 $$\begin{aligned}{} & {} p_T(x_{n+1}|x_{n},a) \propto p_G\left( \mathcal {G}\circ \mathcal {F}(x_n)=x^\text {N1}\right) \nonumber \\{} & {} \quad \cdot p_{CN}\left( x_{n+1}=\mathcal {C}\circ \mathcal {N}\left( x^\text {N1}\right) \right) \end{aligned}$$

for $x_{n+1}$ with its $\theta$-coordinate value being the same as that of $x^F_{n+1}=\mathcal {F}(x_n)$, i.e., for $x^G_{n+1}=\mathcal {G}(x^F_{n+1})=x^\text {N1}$, and

B12 $$\begin{aligned}{} & {} p_T(x_{n+1}|x_{n},a) \propto p_G(\mathcal {G}\circ \mathcal {F}(x_n)=x^\text {N2})\nonumber \\{} & {} \quad \cdot p_{CN}(x_{n+1}=\mathcal {C}\circ \mathcal {N}(x^\text {N2})) \end{aligned}$$

for $x_{n+1}$ with its $\theta$-coordinate value being not the same as that of $x^F_{n+1}=\mathcal {F}(x_n)$ and $x^G_{n+1}=\mathcal {F}(x_n)\pm \delta \theta =x^\text {N2}$. The actual values of the state transition probability are determined so that the equality

B13 $$\begin{aligned} \sum _{x_{n+1}\in \mathcal {X}}p_T(x_{n+1}|x_{n},a)=1 \end{aligned}$$

is hold for each action $a\in \mathcal {A}$. In this way, we prepare a set of $|\mathcal {A}|$ state transition probability matrices of $|\mathcal {X}|\times |\mathcal {X}|$ size, where $|\mathcal {A}|$ and $|\mathcal {X}|$ represent the numbers of element of the sets $\mathcal {A}$ and $\mathcal {X}$, respectively.
